# Cabozantinib Inhibits Growth of Androgen-Sensitive and Castration-Resistant Prostate Cancer and Affects Bone Remodeling

**DOI:** 10.1371/journal.pone.0078881

**Published:** 2013-10-25

**Authors:** Holly M. Nguyen, Nazanin Ruppender, Xiaotun Zhang, Lisha G. Brown, Ted S. Gross, Colm Morrissey, Roman Gulati, Robert L. Vessella, Frauke Schimmoller, Dana T. Aftab, Eva Corey

**Affiliations:** 1 Department of Urology, University of Washington, Seattle, Washington, United States of America; 2 Department of Orthopedics, University of Washington, Seattle, Washngton, United States of America; 3 Biostatistics and Biomathematics Program, Fred Hutchinson Cancer Research Center, Seattle, Washington, United States of America; 4 Exelixis Inc, South San Francisco, California, United States of America; Southern Illinois University School of Medicine, United States of America

## Abstract

Cabozantinib is an inhibitor of multiple receptor tyrosine kinases, including MET and VEGFR2. In a phase II clinical trial in advanced prostate cancer (PCa), cabozantinib treatment improved bone scans in 68% of evaluable patients. Our studies aimed to determine the expression of cabozantinib targets during PCa progression and to evaluate its efficacy in hormone-sensitive and castration-resistant PCa in preclinical models while delineating its effects on tumor and bone. Using immunohistochemistry and tissue microarrays containing normal prostate, primary PCa, and soft tissue and bone metastases, our data show that levels of MET, P-MET, and VEGFR2 are increasing during PCa progression. Our data also show that the expression of cabozantinib targets are particularly pronounced in bone metastases. To evaluate cabozantinib efficacy on PCa growth in the bone environment and in soft tissues we used androgen-sensitive LuCaP 23.1 and castration-resistant C4-2B PCa tumors. *In vivo*, cabozantinib inhibited the growth of PCa in bone as well as growth of subcutaneous tumors. Furthermore, cabozantinib treatment attenuated the bone response to the tumor and resulted in increased normal bone volume. In summary, the expression pattern of cabozantinib targets in primary and castration-resistant metastatic PCa, and its efficacy in two different models of PCa suggest that this agent has a strong potential for the effective treatment of PCa at different stages of the disease.

## Introduction

Metastases remain the major cause of morbidity and mortality in men suffering from advanced prostate cancer (PCa). Despite existing agents that are effective against advanced PCa, survival after development of castration resistance remains very short. Therefore, novel, effective treatments against metastatic and castration-resistant disease are urgently needed.

Cabozantinib is a potent inhibitor of receptor tyrosine kinases, including MET and VEGF receptor 2 (VEGFR2). Other targets inhibited by cabozantinib include AXL, FLT-3, KIT, and RET [1,2]. The effects of cabozantinib have been evaluated in the preclinical setting in multiple cancers, including glioma, breast, lung, and pancreatic cancers. In these studies, cabozantinib reduced tumor invasiveness, proliferation, and angiogenesis while increasing apoptosis [1,2]. Preclinical studies in a model of pancreatic neuroendocrine cancer have provided some insight into the mechanisms of cabozantinib action, suggesting a functional balance between MET and VEGFR2 through involvement of HIF1A [2-5]. However, mechanisms involving other targets of cabozantinib, such as RET, an important target in medullary thyroid carcinoma [6,7], and AXL or KIT, have not been extensively examined or reported. Given the roles of these kinases in tumor biology, cabozantinib inhibition of any or all of these targets may be beneficial for the treatment of PCa by attacking tumor cells on multiple fronts. This type of attack could potentially target effectively heterogeneous cell populations, such as those of PCa. 

Cabozantinib was recently approved by the FDA for the clinical treatment of progressive, metastatic medullary thyroid cancer. This approval followed early observations of cabozantinib activity against this disease in the initial phase I clinical study [5]. Cabozantinib has also demonstrated encouraging results in patients with metastatic, castration-resistant PCa (CRPC) in a phase II adaptive randomized discontinuation trial. Substantial improvements in bone scans were observed in 68% of evaluable patients. Furthermore, 72% exhibited regression in soft tissue lesions, and 67% experienced an improvement in bone pain [3]. However, it is important to note that at 12 weeks the objective response rate was 5%, and 75% of patients showed stable disease [3]. Nevertheless, no other agent has shown this constellation of effects in men with CRPC, indicating a potentially unique mechanism of action for cabozantinib in this disease setting. 

MET and its ligand, hepatocyte growth factor (HGF), have been implicated in the progression of many cancers. MET signaling promotes cell survival, proliferation, invasion, metastasis, and angiogenesis *in vivo* and *in vitro* [8]. In PCa, MET is expressed in primary PCa, and higher levels of expression are detected in PCa metastases in bone [9-11]. Furthermore, MET expression was associated with PCa grade, Gleason score, and poor prognosis [9], and elevated plasma levels of HGF were found to be a poor prognostic indicator in PCa patients [12]. In addition, preclinical results have demonstrated crosstalk between the androgen receptor (AR) and MET signaling, with AR signaling resulting in direct inhibition of MET expression [13,14]. Therefore, upregulation of MET signaling may be associated with the progression of PCa to castration resistance. Accordingly, multiple novel MET inhibitors are being developed against various cancers, including PCa [15]. However, a review of the current literature showed mixed results of MET inhibition in PCa. The MET inhibitors PHA-665752 and PF2341066 inhibited growth of PCa cells [16], and knockdown of MET expression by an adenovirus inhibited PCa growth and lymph node (LN) metastases [17]. In contrast BMS-777607, another MET inhibitor, did not significantly affect the proliferation of PCa cells but inhibited their invasiveness and migration [18]. In the clinic, a number of agents that selectively target MET have failed to show a substantive clinical benefit in patients with CRPC [19,20]. 

VEGFR2 also plays important roles in multiple cancers, including PCa. VEGFR signaling is central in the regulation of angiogenesis and is increased in metastatic CRPC lesions [21]. Furthermore, elevated plasma and urine levels of VEGF are associated with a poor prognosis in PCa [22,23]. Therefore, VEGF/VEGFR2 signaling has been a target for new cancer therapies. Inhibition of the VEGF pathway decreased tumor growth and vasculature in a model of pancreatic islet cancer, though this effect was not sustained and tumors eventually recurred [24]. A clinical trial of bevacizumab, a recombinant anti-VEGF monoclonal antibody, combined with chemotherapy showed increased progression-free survival compared to placebo plus chemotherapy. However, this treatment combination did not significantly improve overall survival compared to the control arm [25]. The limited responses and acquired resistance to anti-VEGF therapy suggest that while angiogenesis through VEGF is an important target for cancer therapies, the development of successful new drugs will require a deeper understanding of factors that facilitate escape from anti-angiogenic therapy and allow for continued tumor survival and vascularization. With this in mind, increases in MET activity have been detected in response to anti-angiogenic therapy and hypoxia [26,27]. Continued administration of the VEGF inhibitor sunitinib increased HGF expression in a murine tumor model, and sunitinib was ineffective when HGF was co-administered [28,29]. In addition, VEGF has been shown to directly activate MET signaling via neuropilin-1 in PCa independent of HGF [11]. These results demonstrate a synergistic action between the VEGF and MET signaling pathways and suggest that a therapy targeting both of these pathways, such as cabozantinib, might be highly relevant in advanced PCa. 

We aimed to determine the expression levels of cabozantinib’s primary targets in advanced PCa and assess its effects on subcutaneous PCa tumors and those growing in bone, specifically with regards to tumor burden and bone turnover. Our results show that the targets of cabozantinib are expressed in PCa metastases and that cabozantinib inhibits tumor growth in bone and soft tissue and exhibits beneficial effects on bone in both intact and castrated male mice. 

## Materials and Methods

All human tissues were obtained from patients who signed written informed consent and upon University of Washington IRB approval. All animal studies described in this manuscript were approved by and performed in compliance with the University of Washington Institutional Animal Care and Use Committee and NIH guidelines.

### Immunohistochemistry (IHC)

Tissue microarrays (TMAs) were used to determine MET, P-MET, and VEGFR2 immunoreactivity in normal prostate (NP) and PCa: 1) UWTMA48: NP and benign prostatic hyperplasia (60 tissues, two cores per tissue) and primary PCa (61 tissues, two cores per tissue); 2) UWTMA52: matched NP and PCa from recurrent and non-recurrent patients (63 recurrent and 64 non-recurrent patients, two cores for each NP and PCa); 3) UWTMA21: PCa metastases from 44 patients (40 bone metastases; 19 liver metastases; 27 LN metastases; and 7 other soft tissue metastases, two cores per tissue); and 4) UWTMA48: 24 LuCaP PCa xenograft models from intact, castrated and docetaxel-treated animals (three cores per tissue). IHC was performed using standard procedures with antigen retrieval [30]. A mouse monoclonal anti-MET antibody (gift from Dr. Knudsen [31]), a rabbit monoclonal anti-P-MET antibody (Cell Signaling, Boston, MA), and a rabbit polyclonal anti-VEGFR2 antibody (Cell Signaling, 55B11, Boston, MA) were used. Specific immunostaining was assessed by a pathologist (XZ) using a three point scale: 2=intense, 1=faint, and 0=absent, and the percentage of cells at each intensity was estimated. 

### Statistical analysis of IHC

For each core in each TMA, a staining index was constructed as a weighted combination of the 3-point staining intensities, with weights given by the percentage of tissue staining at each intensity; see Methods S1. The calculated staining index is a value in the interval [0, 1], where 1 means 100% of cells stained intensively. Linear mixed models were fit to the staining index conditional on the metastasis location with random effects for each patient or animal. Following evaluation of modeling assumptions and data transformation as necessary, the fitted models were used to quantify differences in the immunoreactivity between metastatic locations and test statistical significance. Graphical profiles illustrating the distributions of staining intensity were constructed by calculating simple averages across all non-missing sections in each staining category. Associations between the expression of proteins and clinical parameters were evaluated using linear regression models, and associations with time to PSA recurrence were evaluated using frailty (random effects Cox proportional hazards) models.

### Cell lines

C4-2B cells (Urocor, Inc., Oklahoma City, OK) and MC3T3 cells (ATCC, Manassas, VA) were maintained under standard tissue culture conditions. C4-2B cells were grown in RPMI 1640 and 10 % fetal bovine serum (FBS), and MC3T3 cells were grown in DMEM with 10% FBS. The LuCaP 23.1 PCa xenograft was maintained and serially passaged in CB17 SCID mice [32].

### Animal studies

#### Study 1: Intratibial LuCaP 23.1, 60 mg/kg cabozantinib

Intact six-week-old male beige SCID mice (Charles River, Wilmington, MA) were injected with a LuCaP 23.1 single-cell suspension into the right proximal tibiae as published previously [33]. 200,000 LuCaP 23.1 cells were injected in 20 µL of RPMI 1640 into the right proximal tibia. Animals were randomized into a control group (n=5) or cabozantinib group (n=5) when the serum PSA levels reached detectable levels (0.6 ng/mL, AxSYM Total PSA assay, Abbott Laboratories, Abbot Park, IL). Cabozantinib was dissolved in H_2_O and administered by oral gavage at 60 mg/kg, five times a week for six weeks. Control animals received gavage with H_2_O only. Serum PSA levels and body weights were measured weekly. After sacrifice, tumored tibiae and normal contralateral tibiae were collected and processed for analyses.

#### Study 2: Intratibial C4-2B, 60 mg/kg cabozantinib

The study design was the same as for study 1, except that animals were castrated and C4-2B cells were injected into tibiae two weeks post-castration [34]. C4-2B cells were harvested when ~50% confluent, and 200,000 cells were injected in each tibia. Animals were randomized into a control group (n=11) or a cabozantinib group (n=10). 

#### Study 3: Subcutaneous C4-2B, 60 mg/kg cabozantinib

C4-2B (2x10^6^, 1:1 with Matrigel) cells were injected subcutaneously into castrated male mice. Animals were randomized into a control group (n=8) or a cabozantinib group (n=12) when the tumor volume reached 100 mm^3^. 60 mg/kg cabozantinib was administered by oral gavage five times a week for up to nine weeks. Animals were sacrificed when tumors reached 1000 mm^3^ or when animals were compromised. 

#### Study 4: Intratibial LuCaP 23.1, 30 mg/kg cabozantinib

The study design was the same as in study 1, except that a lower dose of cabozantinib (30 mg/kg) was used and the treatment lasted up to 15 weeks. Animals were randomized into a control group (n=10) or a cabozantinib group (n=10).

### Micro-CT

A Scanco vivaCT 40 high-resolution µCT scanner was used to analyze a 0.85 mm section spanning the proximal tibia metaphysis of tumored and non-tumored contralateral tibiae (LuCaP 23.1: n=3–5; C4-2B: n=5–10 per group). Bone volume (BV), tissue volume (TV), trabecular separation (Tb.Sp), trabecular thickness (Tb.Th), and trabecular number (Tb.N) were determined and the BV/TV was calculated. 

### Statistical analyses of animal studies

Longitudinal tumor measurements and PSA serum levels were log-transformed and modeled using linear mixed models conditional on the treatment group with random effects for each animal; see Methods S1. Following a standard diagnostic assessment of model fit, we simulated 1000 datasets from each ﬁtted model, calculated the empirical mean and 95% confidence limits at each time point, and reﬁt the models to these datasets. The final results represent means and 95% conﬁdence limits of 1000 bootstrap replicates. Following standard checks of model assumptions, 2-sided t-tests were applied to test for differences in serum PSA, tumor volume, body weight, and IHC staining index levels. The statistical significance of the differences in bone parameters was determined using a 2-sided t-test.

### RT-PCR analysis

RNA extraction, cDNA synthesis, and qPCR were performed as described previously [35]. The primers and annealing conditions are listed in Table S1. RNA was extracted from subcutaneous tumors. Relative expression of the target messages was determined based on four-fold dilution of the calibrator cDNAs. We used LNCaP cDNA for RET; PC-3 cDNA for MET, AXL and KIT, and LuCaP 23.1 cDNA for murine VEGFR2 ( VEGFR2m). Specific signals were normalized to RPL13a levels.

### In vitro experiments

The effects of cabozantinib on proliferation, mineralization, alkaline phosphatase (ALP) activity, and AR transcriptional activity were assessed *in vitro* as described previously [36]; see Methods S1.

## Results

### Levels of MET, P-MET and VEGFR2 during PCa progression

To address whether cabozantinib targets are expressed in PCa, we evaluated the levels of MET, P-MET, and VEGFR2 in tissues representing normal prostate and different stages of PCa progression. 

#### NP vs primary PCa

MET and P-MET: Our results showed that MET is present at high levels in the NP and primary PCa cells, but there was only marginal evidence of differences between these tissues (mean staining index NP: 0.96, 95% CI 0.93–0.98; PCa: 0.92, 95% CI 0.88–0.96; P=0.06); see Figure S1. Despite the high levels of MET in NP and PCa, minimal immunoreactivity of P-MET was detected in these tissues, and there was no evidence of differences between these tissues (mean staining index NP: 0.16, 95% CI 0.00–0.32; PCa: 0.11; 95% CI 0.00–0.27; P=0.49); see Figure S1. There was no evidence that MET or P-MET immunoreactivity was associated with risk of biochemical recurrence after controlling for age, Gleason sum, and tumor volume.

VEGFR2: Normal prostate epithelial cells and PCa cells both exhibited low VEGFR2 immunoreactivity, and there was marginal evidence of higher VEGFR2 in PCa vs NP (mean staining index NP: 0.03, 95% CI 0.00–0.05; PCa: 0.07, 95% CI 0.03–0.11; P=0.02); see Figure S1. Strong staining was present in the stroma and vasculature of both NP and PCa. Similar to MET, there was no evidence that VEGFR2 expression was associated with risk of PSA recurrence after controlling for age, Gleason sum, and tumor volume. 

#### Primary PCa vs metastases

**Figure 1:**
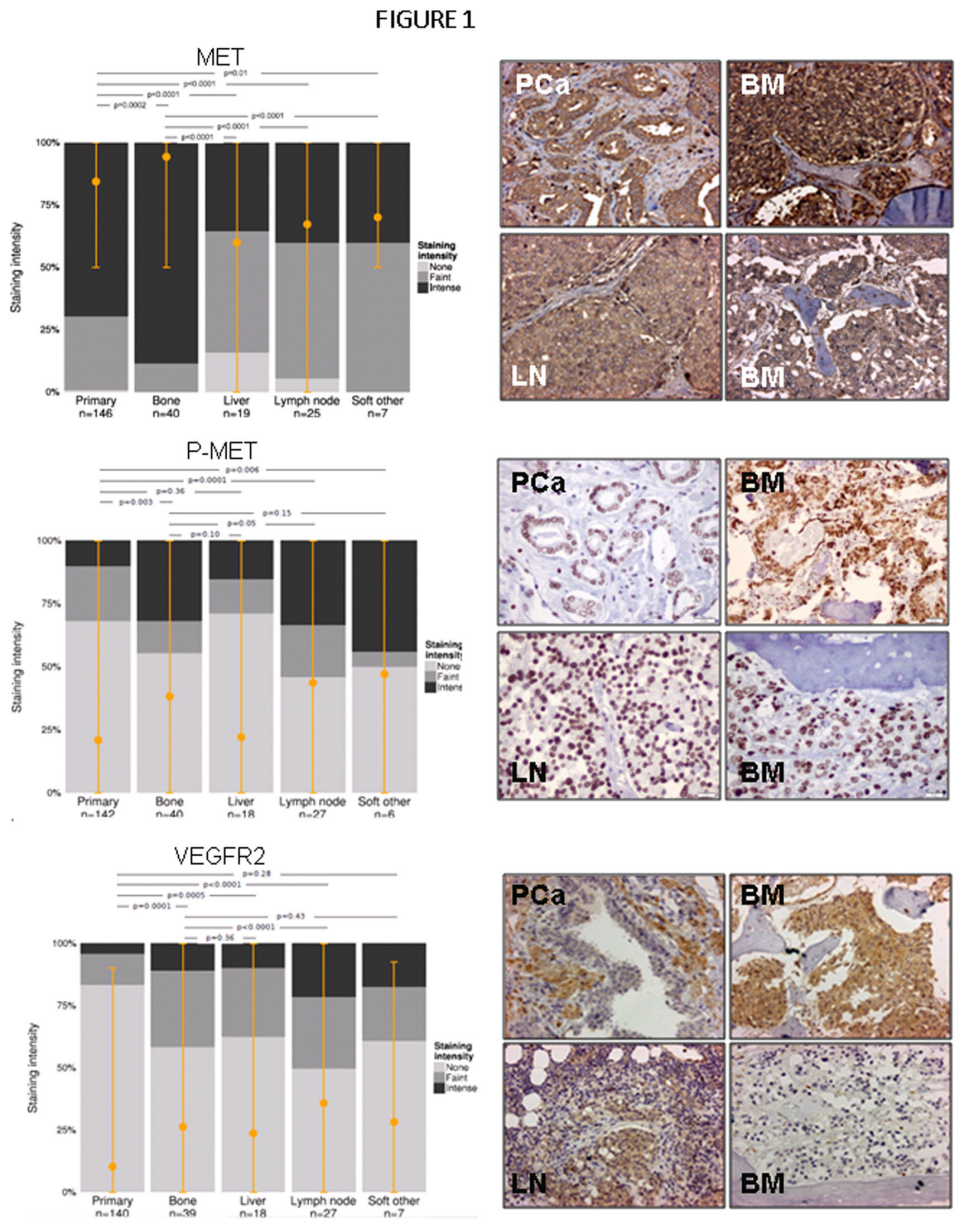
Expression of MET, P-MET, and VEGFR2 in primary and metastatic patient samples. IHC and analyses were performed as described in the Methods section. Graphical profiles illustrating distributions of staining intensity were constructed by calculating simple averages across all non-missing sections in each staining category. In each site, the mean staining index is marked by a filled orange circle and orange bars represent 95% CIs. Representative examples of staining are shown for each protein. A. MET is strongly expressed in both primary and metastatic PCa, though it is significantly increased in BM and decreased in soft tissue metastases vs. primary PCa. B. P-MET levels are higher in BM, LN and other soft tissue metastases, while no alteration was detected in liver metastases when compared to primary PCa. C. VEGFR2 expression is significantly increased across PCa metastatic lesions as compared to primary PCa. Images were taken at 400 x magnification.

Because of the reported increases of MET in PCa metastases [12], we set out to evaluate whether this increase can be detected in tissues from our cohort of patients. For this comparison, we used the results from metastases on TMA 21 and combined results of the PCa tissues from UWTMA48 and UWTMA52 (127 patients). MET was detected in PCa metastases with very strong evidence of higher immunoreactivity staining in bone metastases (BM) compared to primary PCa (mean staining index primary PCa: 0.83, 95% CI 0.82–0.87; BM: 0.94, 95% CI 0.89–0.98; P=0.0002); see Figure 1. In contrast, MET levels were significantly lower in all soft tissue metastases compared to primary PCa (mean staining index liver: 0.70, 95% CI 0.62–0.77, P<0.0001; LN: 0.78, 95% CI 0.71–0.83, P<0.0001; other soft: 0.79, 95% CI 0.67–0.93, P=0.01).

P-MET was detected in PCa metastases with strong evidence of higher immunoreactivity in BM compared to primary PCa (mean staining index primary PCa: 0.22, 95% CI 0.17–0.26; BM: 0.37, 95% CI 0.30–0.45; P=0.003); see Figure 1. P-MET levels were also higher in all soft tissue metastases compared to primary PCa, though not all differences were significant (mean staining index liver: 0.28, 95% CI 0.13–0.38, P=0.35; LN: 0.46, 95% CI 0.35–0.56, P=0.0001; other soft: 0.51, 95% CI 0.27–0.70, P=0.006).

VEGFR2 was detected in PCa metastases with very strong evidence of higher immunoreactivity in BM compared to primary PCa (mean staining index primary PCa: 0.10, 95% CI 0.07–0.14; BM: 0.25, 95% CI 0.19–0.31; P=0.0001); see Figure 1. The levels of VEGFR2 were also higher in all soft tissue metastases compared to primary PCa, though not all differences were significant (mean staining index liver: 0.29, 95% CI 0.17–0.38, P=0.0005; LN: 0.40, 95% CI 0.29–0.46, P<0.0001; other soft: 0.19, 95% CI 0.04–0.38, P=0.28).

#### PCa metastases

Since the microenvironment influences the gene expression profiles of tumor cells, we also analyzed the MET expression levels by metastatic site. The levels of MET were significantly higher in BM in comparison to liver, LN, and other soft tissue metastases (mean staining index BM: 0.94, 95% CI 0.91–0.98; mean staining index liver: 0.59, 95% CI 0.52–0.66, P<0.0001; LN: 0.67, 95% CI 0.61–0.73, P<0.0001; other soft: 0.68, 95% CI 0.57–0.83, P<0.0001).

The levels of P-MET were weakly or non-significantly different in BM in comparison to liver, LN, and other soft tissue metastases (mean staining index BM: 0.37, 95% CI 0.32–0.44; liver: 0.28, 95% CI 0.13–0.36, P=0.10; LN: 0.46, 95% CI 0.34–0.53, P=0.05; soft other: 51%, 95% CI 0.27–0.69); see Figure 1. However, due to different processing of BM and soft tissue metastases (BM processing requires decalcification) and low stability of phosphorylation under acidic conditions, the actual levels of P-MET might actually be higher in the BM than our data indicate. 

The VEGFR2 levels were significantly different in BM in comparison to LN metastases (mean staining index BM: 0.25, 95% CI 0.21-0.31; LN: 0.40, 95% CI 0.30-0.45, P<0.0001) but not the liver or other soft tissue metastases. 

#### Associations between the MET, P-MET, and VEGFR2 staining index and AR, PSA, and PSMA

Crosstalk between AR, MET and VEGFR2 signaling has been reported in CRPC (13,14). Therefore, we evaluated associations between MET, P-MET and VEGFR2 staining (determined in this study) and AR, PSA, and PSMA staining (from historical data) in metastases. Our analysis, which was based on a linear mixed model (see Methods S1), did not detect any significant associations between any of the pairs of the selected proteins in BM or soft tissue metastases (data not shown). 

### Cabozantinib targets in PCa xenografts

#### qPCR

To determine the expression of selected cabozantinib targets in PCa, we evaluated levels of MET, murine VEGFR2, AXL, KIT, and RET mRNA in 24 different LuCaP PCa xenografts that closely model the heterogeneity of PCa in humans [37]. Our qPCR results show that all cabozantinib targets are expressed in these models at varying levels; see Figure S2A. Stratifying the models to neuroendocrine tumors (NE) and adenocarcinoma (AD) revealed high to moderate evidence of higher levels of all targets in NE LuCaP models (n=4) as compared to AD models (n=20) see Figure S2B. Since crosstalk between AR, MET, and VEGFR2 signaling has been reported, we also examined whether the expression of cabozantinib targets correlates with AR expression or responses to castration. To this end, we classified xenografts as “highly responsive to castration” if castration resulted in a more than 3-fold survival benefit. Our analyses did not reveal any significant associations between the levels of AR and the cabozantinib targets. Furthermore, while the mean mRNA levels of cabozantinib targets were higher in models that do not respond well to castration, these differences did not reach significance.

#### IHC

To gain a better understanding of cabozantinib’s potential effects in patients with advanced CRPC who are on ADT and/or treated with docetaxel, we examined the levels of MET, P-MET, and VEGFR2 in LuCaP tumors from intact, castrated, and docetaxel-treated animals. Our analyses show moderate evidence that MET and P-MET expression levels are negatively correlated across tumor types (R=0.29; P=0.02), and marginal evidence that the mean MET and P-MET staining indices are 5–6% higher in tumors after docetaxel treatment than in tumors from intact animals (P=0.08 and P=0.05, respectively). Our analyses did not reveal any evidence that expression levels for any other pair of proteins are correlated across or within tumor types (all P>0.14), that mean MET and P-MET staining indices are different between tumors harvested from intact and castrate animals, or that mean MET and P-MET staining indices differ significantly between LuCaP xenografts that display a high and low response to castration; see Figure S2C. VEGFR2 did not show any significant immunoreactivity in tumor cells in our models. 

### Preclinical Efficacy Studies

To increase our understanding of cabozantinib’s effects on PCa bone metastases, we examined its effects on serum PSA, body weight, and bone turnover in models of PCa growth in the bone. Similarly, we assessed changes in serum PSA, tumor volume, and body weight in response to treatment in animals bearing subcutaneous tumors.

#### Cabozantinib inhibits tumor growth in bone

**Figure 2:**
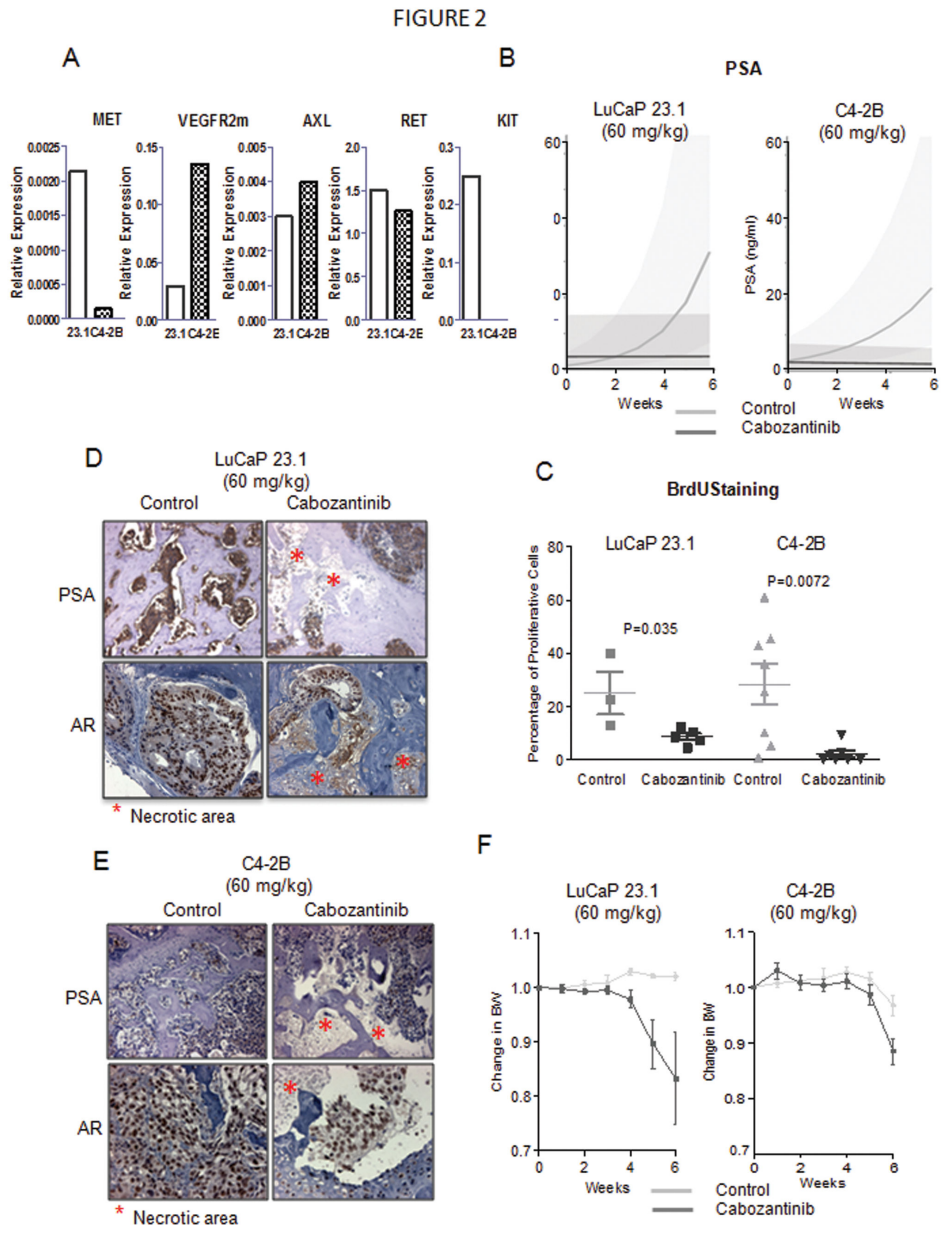
Cabozantinib (60 mg/kg) inhibits tumor growth in androgen-sensitive and castration-resistant PCa in bone. A. Levels of cabozantinib receptors in LuCaP 23.1 and C4-2B subcutaneous tumors. qPCR was used on RNA isolated from subcutaneous tumors to determine expression levels of MET, VEGFR2m, AXL, RET and KIT. To calibrate the signal we used four-fold dilution of LNCaP cDNA (RET), PC-3 cDNA (MET, AXL, KIT) and LuCaP 23.1 (VEGFR2m). Selection of the calibrator cDNA was based on signal for each specific message. Signal was normalized to housekeeping gene RPL13a. Our results indicate that LuCaP 23.1 tumors express all of the cabozantinib targets and C4-2B tumors express VEGFR2m, AXL and RET, low levels of MET, and no KIT. In these qPCR experiments, we measured relative levels of the target transcripts and not their actual numbers, therefore we cannot compare expression levels of the different targets to each other, and comment whether RET, which gave the higher signal, might be expressed at higher copy number vs MET, and therefore is more important in these models. B. Linear models of PSA growth show that cabozantinib decreases PSA levels in both androgen-sensitive LuCaP 23.1 and castration-resistant C4-2B models. C. BrdU staining of tibiae shows that cabozantinib decreases proliferation of androgen-sensitive and castration-resistant tumor cells in bone, 2-sided t-test was used to determine the significance of the differences. D & E. AR and PSA immunoreactivity is present in control tumors. Cabozantinib treatment resulted in decreases in AR and PSA immunoreactivity in both models (LuCaP 23.1 (D) and C4-2B (E)). C4-2B cells express lower levels of PSA in comparison to LuCaP 23.1, and the staining is weaker in these tumor. Furthermore, large necrotic areas of tumor are present in the treated tibiae (marked by red asterics). F. 60 mg/kg cabozantinib is well tolerated up to 4 weeks in androgen-sensitive LuCaP 23.1 animals. After this period significant BW decreases vs. control were detected (up to 17%), but because of variation and number of animals, these decreases did not reach significance. 60 mg/kg cabozantinib is well tolerated up to 5 weeks in the castration-resistant C4-2B model, with a 12% significant decrease at week 6. Significance was determined by comparing enrollment BW to BW at each week using 2-sided t-test. Mean ± SEM of the groups is plotted.

To evaluate the efficacy of cabozantinib on growth of PCa in bone, we treated intact or castrate animals bearing intratibial LuCaP 23.1 or C4-2B tumors, respectively. We selected these two models because LuCaP 23.1 elicits a pronounced osteoblastic reaction and C4-2B elicits a mixed osteoblastic/osteolytic response. Furthermore, LuCaP 23.1 represents androgen-sensitive PCa while C4-2B represents castration-resistant disease. Our qPCR results show that MET, VEGFR2m, KIT, RET and AXL are expressed in LuCaP 23.1. In C4-2B tumors we detected VEGFR2m, AXL and RET, very low levels of MET and no signal for KIT (results are shown in Figure 2A). Expression of these receptors in LuCaP 23.1 and C4-2B tumors support the hypothesis that cabozantinib will alter the biology of these tumors. Cabozantinib (60 mg/kg) inhibited the growth of both tumors in bone as demonstrated by decreases in serum PSA levels; see Figure 2B. Weekly changes in serum PSA were significantly different between the control and cabozantinib groups in the LuCaP 23.1 model (P<0.0001), where PSA increased by 76% per week in the control group but by 0.2% per week in the cabozantinib group. PSA changes were also significantly different between the control and cabozantinib groups in the C4-2B model (P=0.0066), where PSA increased by 35% per week in the control group but decreased by 2.9% per week in the cabozantinib group. Cabozantinib also inhibited proliferation of the remaining viable cells in the tumors based on BrdU staining; see Figure 2C. The inhibition of tumor progression was also noticeable when we evaluated AR and PSA immunoreactivity; LuCaP 23.1 and C4-2B tumors from animals treated with cabozantinib showed less intense AR and PSA staining in the remaining tumor cells as well as large necrotic areas; see Figure 2D & E. C4-2B cells express lower levels of PSA as seen in Figure 2B and that is also reflected in much lower PSA immunoreactivity in these tumor cells. 

#### Cabozantinib alters bone remodeling in tumored bone

**Figure 3:**
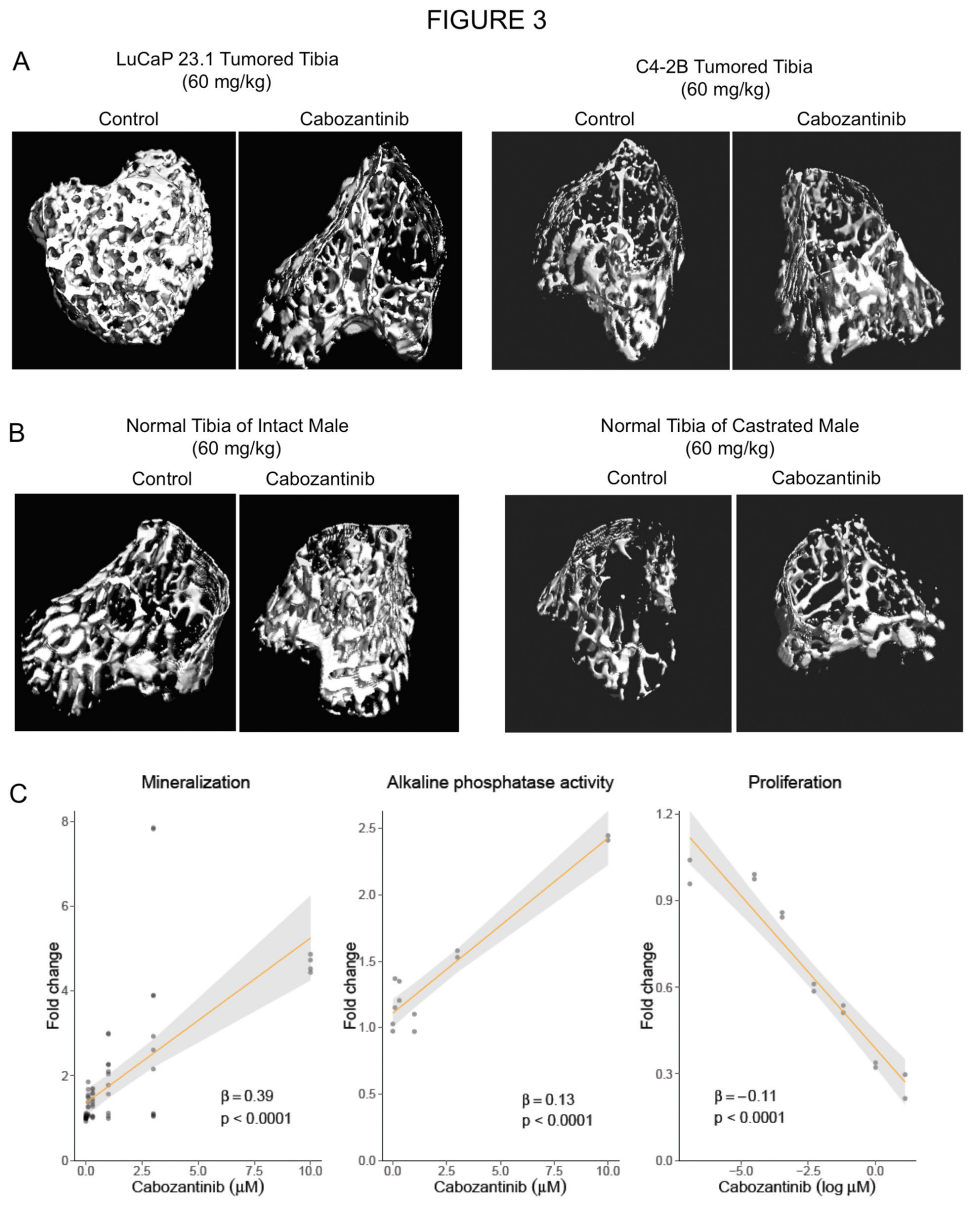
Cabozantinib attenuates bone responses to tumor and increases normal bone volume in tumor unaffected areas. A. LuCaP 23.1 and C4-2B cell growth in tibiae causes large increases in trabecular bone volume. µCT images show that cabozantinib alleviates the bone response to both tumors. In LuCaP 23.1 tumored tibiae cabozantinib caused decreases in BV, while increases in BV were detected in C4-2B tumored tibiae of cabozantinib-treated animals vs control-tumored tibiae. The overall effects are combination of abolishment of tumor effects on the bone as well as cabozantinib effects on normal bone. Details of the effects are provided in Table 1. B. Analysis of non-tumored contralateral tibiae of the experimental animals shows that treatment with cabozantinib results in increased bone volume in both intact and castrated male mice. C. *In* *vitro*, cabozantinib treatment inhibits proliferation of MC3T3 pre-osteoblast cells in a concentration-dependent manner, while promoting ALP activity and mineralization. Fold change in cells response measures was estimated from a single experiment that was repeated three times, and association with cabozantinib concentration was quantified and tested using linear regression models.

We performed a detailed analysis of cabozantinib’s effects on the bone/tumor microenvironment by µCT. We have chosen this type of analysis instead of histomorphometry analysis because µCT evaluates the whole tibia in 3D while histomorphometry analyses are done usually on a single 2D longitudal section of the tibia. The analysis of trabecular bone showed that LuCaP 23.1 growth results in significant increases in bone volume (tumored tibiae: 0.42 ± 0.06 (mean ± SEM); normal tibiae: 0.09 ± 0.01; 5-fold increase in BV/TV, P=0.02). These increases were attenuated by cabozantinib, resulting in a 52% decrease in BV/TV as compared to the control LuCaP 23.1 tibiae. This decrease was reflected in altered trabecular number (Tb.N), trabecular thickness (Tb.Th), and trabecular separation (Tb.Sp); see Table 1. C4-2B growth in bone also resulted in moderate evidence of BV/TV increases (40% increase, P=0.06). However, in contrast to the LuCaP 23.1 model, cabozantinib treatment resulted in a trend toward increased bone volume in the C4-2B model, as evidenced by a 58% increase in BV/TV (P=0.08); see Table 1. Representative examples of µCT are presented in Figure 3A.

**Table 1:** Micro CT Analysis of Cabozantinib Effects on Tumored and Normal Bone.

TUMORED TIBIAE									
		**LuCaP 23.1**	***P***		**LuCaP 23.1**	***P***		**C4-2B**	***P***
		**60 mg/kg**			**30 mg/kg**			**60 mg/kg**	
**BV/TV**	**Control**	0.42 ± 0.06	*0.0056*	**Control**	0.71 ± 0.05	*0.0000*	**Control**	0.08 ± 0.02	*0.0765*
	**Cabo**	0.20 ± 0.04		**Cabo**	0.19 ± 0.08		**Cabo**	0.12 ±0.05	
	**Change**	**-52.4%**		**Change**	**-73.2%**		**Change**	**+ 58.1%**	
**Tb.N**	**Control**	6.30 ± 0.28	*0.0010*	**Control**	6.06 ± 0.83	*0.0002*	**Control**	3.28 ± 0.71	*0.6280*
	**Cabo**	3.93 ± 0.39		**Cabo**	2.05 ± 0.53		**Cabo**	3.61 ± 1.35	
	**Change**	**-37.6%**		**Change**	**-48.8%**		**Change**	**+9.0%**	
**Tb.Th**	**Control**	0.09 ± 0.01	*0.2849*	**Control**	0.17 ± 0.02	*0.0001*	**Control**	0.05 ± 0.00	*0.0115*
	**Cabo**	0.09 ± 0.01		**Cabo**	0.11 ± 0.01		**Cabo**	0.06 ± 0.01	
	**Change**	**-6.9%**		**Change**	**-34.4%**		**Change**	**+14.9%**	
**Tb.Sp**	**Control**	0.14 ± 0.01	*0.0024*	**Control**	0.12 ± 0.03	*0.0002*	**Control**	0.32 ± 0.09	*0.7836*
	**Cabo**	0.25 ± 0.03		**Cabo**	0.33 ± 0.06		**Cabo**	0.31 ± 0.10	
	**Change**	**+78.6%**		**Change**	**+175.0%**		**Change**	**-3.1%**	
NORMAL TIBIAE									
		**intact male**	***P***		**castrated male**	***P***			
		**60 mg/kg**			**60 mg/kg**				
**BV/TV**	**Control**	0.088 ± 0.014	*0.0299*	**Control**	0.055 ± 0.007	*0.0092*			
	**Cabo**	0.164 ± 0.037		**Cabo**	0.075 ± 0.009				
	**Change**	**+86%**		**Change**	**+36.2%**				
**Tb.N**	**Control**	3.185 ± 0.382	*0.0074*	**Control**	2.820 ± 0.316	*0.0643*			
	**Cabo**	4.424 ± 0.194		**Cabo**	3.221 ± 0.221				
	**Change**	**+38.9%**		**Change**	**+14.2%**				
Tb.Th	**Control**	0.051± 0.003	*0.0741*	**Control**	0.046± 0.004	*0.6866*			
	**Cabo**	0.0618 ± 0.007		**Cabo**	0.047 ± 0.005				
	**Change**	**+21.9%**		**Change**	**+2.9%**				
**Tb.Sp**	**Control**	0.333± 0.037	*0.0075*	**Control**	0.365 ± 0.043	*0.1647*			
	**Cabo**	0.217 ± 0.015		**Cabo**	0.319 ± 0.017				
	**Change**	**-34.8%**		**Change**	**-12.6%**				

BV/TV: Bone volume in tissue volume; Tb.N: number of trabeculae; Tb.Th.: trabecular separation; Tb.Sp. trabecular separation

#### Cabozantinib alters bone remodeling in normal bone

To evaluate the effects of cabozantinib on non-tumored bone, we analyzed the contralateral non-tumored tibiae of the treated and control animals by µCT. Our analyses show that cabozantinib treatment increased BV/TV in intact and castrated male mice; see TABLE 1. Representative examples of µCT images are shown in Figure 3B.

#### Cabozantinib affects on body weight

We also monitored the effects of this treatment on body weight to evaluate its tolerability. A 60 mg/kg dose of cabozantinib in animals bearing LuCaP 23.1 tumors was well tolerated for 4 weeks with a maximum body weight decrease of only 5.4%. Larger decreases were detectable at week 5 and 6 (14% and 22%, respectively), but none of these differences reached statistical significance. Similar results were observed in animals with C4-2B tumors, with decreases of 5.5% at 4 and 5 weeks, and 2.7% at week 6, which were also not statistically significant; see Figure 2E. 

#### Effects of 30 mg/kg cabozantinib on tumor growth in bone

**Figure 4:**
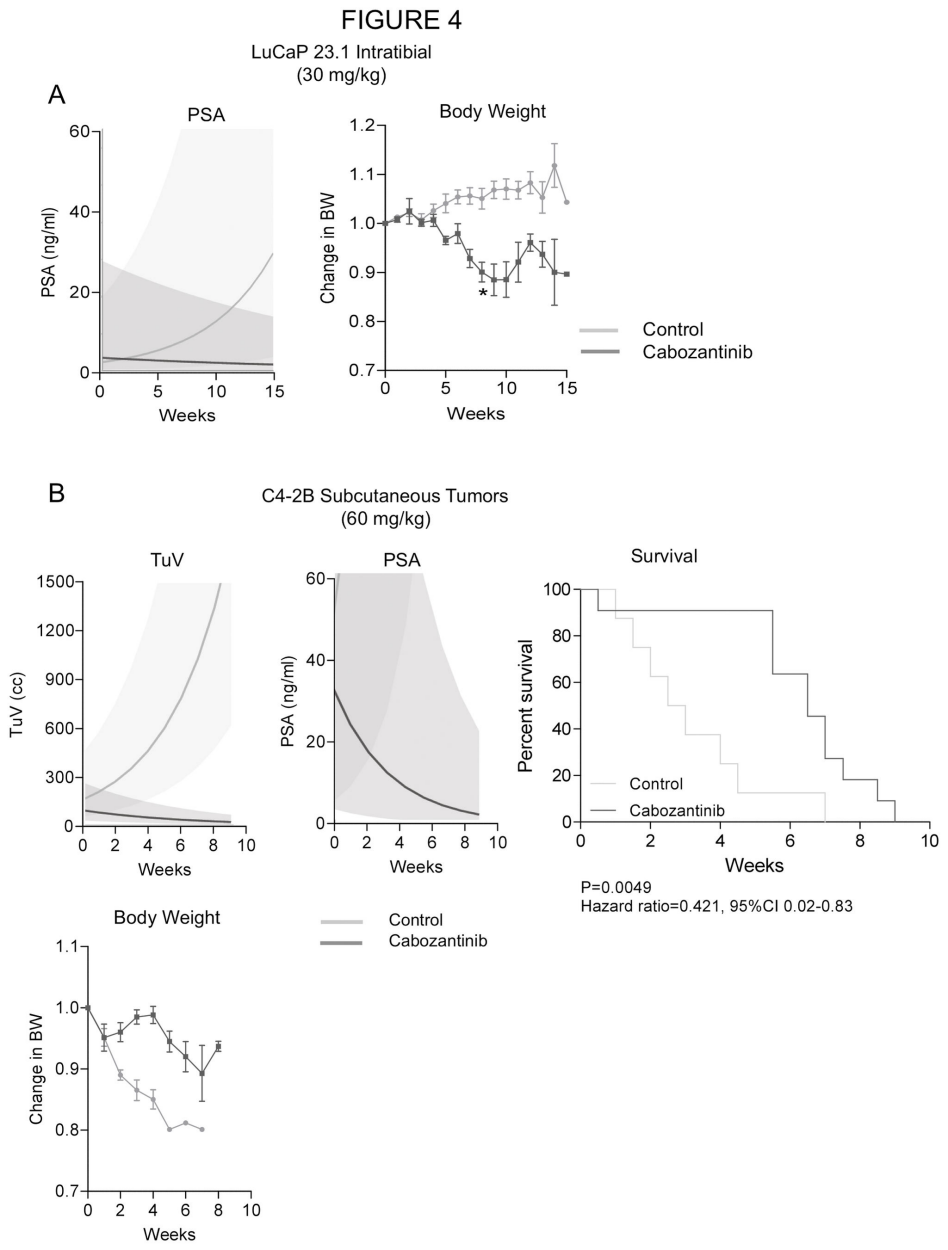
A. A lower dose of cabozantinib at 30 mg/kg also inhibits tumor progression as demonstrated by decreases in serum PSA in animals bearing LuCaP 23.1 tumors in the tibiae. The lower dose was well tolerated for 6 weeks of treatment with no significant body weight loss. Prolonged treatment (7–15 weeks) caused 7–11% body weight decreases which were, however, statistically significant (P=0.0002-0.04). Significance of the changes was determined by comparing enrollment BW to BW at each week using 2-sided t-test. Mean ± SEM is plotted. B. Cabozantinib treatment (60 mg/kg) inhibited tumor growth of subcutaneous castration-resistant C4-2B tumors as determined by TV and serum PSA levels. This treatment also significantly increases survival as determined by log-rank test. C4-2B tumor growth causes decreases of BW in the experimental animals (4–13%, P= 0.0001-0.003), and the cabozantinib treatment prevents this effect resulting in no significant BW loss. Significance of the changes was determined by comparing enrollment BW to BW at each week using 2-sided t-test. Mean ± SEM of the group is plotted.

Because of the above described decreases in body weight after prolonged treatment, we evaluated whether a lower dose of cabozantinib maintains the tumor inhibitory effects in the bone while alleviating the loss in body weight. For this study, we used LuCaP 23.1 in intact male mice. Our results demonstrate that this lower dose inhibits tumor progression as demonstrated by decreases in serum PSA ; see Figure 4A. Weekly changes in serum PSA were significantly different between the control and cabozantinib groups (P<0.0001); PSA increased by 19% per week in the control group but decreased by 4.6% per week in the cabozantinib group. Furthermore, the lower cabozantinib dose resulted in body weight decreases of only 4% at week 6, which were not statistically significant. Significant decreases of 7–11% in body weight occurred after 8 weeks of cabozantinib treatment (P=0.0002-0.030). 

#### Cabozantinib inhibits growth of subcutaneous tumors

To evaluate the efficacy of cabozantinib in soft tissue metastases, we analyzed tumor progression in a subcutaneous C4-2B model. Cabozantinib treatment resulted in a significant decrease in tumor volume (TuV) and serum PSA in this model. TuV increased by 30% per week in the control group while decreasing by 14% per week in the cabozantinib group (P<0.0001); PSA increased by 71% per week in the control group while decreasing by 28% per week in the cabozantinib group (P<0.0001); see Figure 4B. These data indicate that cabozantinib affects the tumor independently of the bone microenvironment. Looking into the mechanisms of cabozantinib effects we first examined whether cabozantinib treatment altered levels of its receptors in C4-2B subcutaneous tumors. Our results showed a trend to decreased VEGFR2m levels (-63%, P=0.057) with cabozantinib treatment. We hypothesize, that the lower levels of VEGFR2m signal indicate cabozantinib’s inhibition of angiogenesis and resulting lower microvessel density, which leads to lower levels of VEGFR2m messages in our samples. Relative expression of AXL was increased 3 fold after cabozantinib treatment, which might be a negative feedback loop, but because of the large variation between the samples this alteration was not significant. No significant changes were detected in relative expression of MET and RET (Figure S3.). Analysis of additional genes associated with angiogenesis by qPCR showed decreases in HIF1A in the cabozantinib group compared to the control group (~−49%, P=0.054) but not in levels of murine endoglin, another gene associated with vasculature (Figure S3). Based on these results we cannot make final conclusions about the effects of cabozantinib on angiogenesis. However, decreases in VEGFR2m point in the direction of cabozantinib modifying the tumor microenvironment (one should note that the PCR analysis had only 80% power to detect large differences between the groups (50-100% differences) due to a large variation between the animals. qPCR evaluation of cabozantinib effects on genes associated with tumor progression revealed decreased levels of MYC (−38% P=0.092), increased levels of the human homolog of endoglin (+185%, P=0.0058) and no alteration in the levels of cyclin D. Evaluating the alteration of genes associated with apoptosis showed a trend to decreased survivin (~-39%, P=0.025), but no alteration in levels of BCL2 and BIM (Figure S3.). A survival analysis of the experimental animals demonstrated a significantly decreased risk of death in animals treated with cabozantinib (P=0.005) over control animals; see Figure 4B.

In contrast to animals with tumors in bone, the animals bearing subcutaneous tumors treated with 60 mg/kg cabozantinib did not show decreases in body weight; in fact, treated animals had higher body weights in comparison to control animals (3–5 weeks, increases 5.5–22%, P<0.005); see Figure 4B. 

### In Vitro Effects of Cabozantinib

Given the pleiotropic effect observed on bone in non-tumored and tumored bone in intact and castrated animals, we evaluated the effects of cabozantinib on osteoblasts and tumor cells directly. In concordance with the *in vivo* effects we detected, our *in vitro* data show that cabozantinib increased mineralization and alkaline phosphatase activity in MC3T3 cells while inhibiting proliferation of these cells; see Figure 3C. Interestingly, our *in vitro* experiments did not show any significant inhibitory effects of cabozantinib (concentration in range 1-10 µM) on the proliferation of C4-2B cells in RPMI1640 supplemented with 10% FBS and on the AR-mediated transcription (data not shown). 

## Discussion

Recent years have seen the development of a variety of new drugs for the treatment of advanced metastatic CRPC that provide modest yet significant improvements in overall patient survival and symptom management. Beyond chemotherapeutics, inhibitors of androgen signaling (e.g. abiraterone and MDV3100), as well as new treatments to minimize skeletal related events (e.g. zoledronic acid and denosumab) have been modestly successful in managing patient symptoms in late stage disease. However, sustained suppression of CRPC, particularly in bone, will likely require new drugs and/or drug combinations that target both the tumor and its interplay with the bone microenvironment. In this context, drugs targeting angiogenic factors and tyrosine kinases have been of particular interest, as both have been shown to influence tumor biology and bone turnover [38,39]. 

Cabozantinib inhibits multiple receptor tyrosine kinases (RTKs) that play key roles in tumor and bone biology as well as in angiogenesis. Therefore, this drug has the potential to affect the malignant tumor cells and the tumor microenvironment. In a phase II randomized discontinuation trial, cabozantinib has shown encouraging results. Sixty-eight percent of patients showed improved bone scans, including complete resolution of lesions in 12 percent of these patients, and regression of soft tissue lesions in 72 percent of evaluable patients. Even though 5% of patients showed an objective response, and 75% of patients showed stable disease at 12 weeks, these results are still encouraging given the heterogeneous nature of PCa and the lack of non-palliative treatments for bone metastases. Furthermore, in 31 patients with stable disease that were randomized to placebo and cabozantinib in the randomized stage, cabozantinib treatment resulted in a median progression-free survival of 23.9 weeks (95% CI 10.7 to 62.4 weeks) compared to 5.9 weeks (95% CI 5.4 to 6.6 weeks, hazard ratio 0.12, P<0.001) with placebo. These results demonstrate the promising clinical activity of cabozantinib in advanced PCa. Little direct evidence to date has shown cabozantinib’s inhibitory effects on the tumor and bone response to tumors, as well as its effects on normal bone *in vivo*, particularly in the context of variable androgen sensitivity. 

To determine the target rationale for cabozantinib in advanced PCa, we first set out to examine the expression of MET and VEGFR2 and levels of P-MET in PCa metastases. Our results highlight MET and VEGFR2 as important targets in PCa metastases, especially in bone, as levels of MET, P-MET and VEGFR2 were significantly upregulated in PCa BM as compared to primary PCa. Furthermore, both of the receptors are also expressed in soft tissue metastases, indicating that MET and VEGFR2 play important roles in the progression of PCa in multiple metastatic settings. In addition, the expression profiles of all cabozantinib targets tested (MET, VEGFR2, AXL, RET, and KIT) in LuCaP PCa xenografts demonstrate that all of these targets are expressed in advanced PCa. The expression pattern in the xenografts is heterogeneous, suggesting that cabozantinib may be broadly applicable in PCa treatment. In concordance with our results with the LuCaP models, AXL, RET and KIT were also detected in advanced PCa in other studies [40-42]. 

In our studies, we focused on advanced metastatic disease but our results also provided additional information about primary disease. Our analyses did not reveal significant differences between MET and P-MET levels in NP epithelium vs. primary PCa, but indicated increases in VEGFR2 in primary PCa. In addition, there was no association between levels of MET, P-MET and VEGFR2 with disease recurrence. Similarly, no differences in MET levels were detected between NP and PCa in another report [10], but marked increases in MET with PCa progression were observed. Furthermore, a correlation with stage and grade was detected by others [9]. Thus, heterogeneity in the expression levels of the various receptors may confound the detection of a potential association.

The AR is important in primary PCa as well as in advanced CRPC. Interestingly, AR activity has been reported to suppress MET expression in preclinical models [13,14], suggesting that MET expression or activity may be tightly linked with AR activity in advanced CRPC. In our study, however, we did not detect any significant associations between MET, P-MET or VEGFR2 with the levels of AR, PSA or PSMA in patient samples. Also, no significant associations between MET or P-MET and AR levels or responses to androgen ablation were revealed in 24 adenocarcinoma LuCaP xenografts examined in our study. Therefore our results do not indicate AR regulation of MET or any association with response to castration. This result agrees with a report showing that expression of MET examined by IHC did not appear to be increased in malignant prostate cells from patients who had undergone androgen ablation therapy compared to those who had not [10]. However, increased expression of MET was detected by qPCR in tumor samples from CRPC patients compared to metastatic non-castrate PCa patients [43]. It is important to note that both of the preclinical studies that report a link between AR signaling and MET employed cell lines in their studies (LNCaP and CWR22) [13,14], while we and those who have reported results in concordance with ours examined patient samples. Studies examining the expression levels of MET and other RTKs in the same patients before and after androgen deprivation and/or transition to castration resistance are needed to more definitively determine the roles and/or relationships of these receptors in the development and progression of primary PCa, as well as in the development of castration resistance. 

Despite no apparent association between AR and MET in clinical specimens, evaluation of the xenografts showed that neuroendocrine LuCaP PCa models express higher levels of all cabozantinib targets in comparison to adenocarcinoma models. This finding indicates that this rare but aggressive subtype of PCa, which is not dependent on AR signaling, may be sensitive to cabozantinib or other agents targeting a similar profile of RTKs. Killing or inhibiting the growth of neuroendocrine cells might also provide an additional benefit in the treatment of adenocarcinoma, since it has been reported that in adenocarcinoma the presence of the rare neuroendocrine cells supports tumor progression. Efficacy of simultaneous inhibition of VEGFR2 and MET was also shown in pancreatic neuroendocrine tumors, with decreased tumor growth and reduction in invasion and metastasis [4].

In the phase II randomized discontinuation trial, cabozantinib treatment resulted in resolution of lesions on bone scan and regression of soft tissue lesions. However, the effects of cabozantinib on PCa tumors in soft tissues or in the bone microenvironment have not yet been delineated. Therefore, we focused on evaluating the effects of cabozantinib on osteoblastic and mixed osteoblastic/osteolytic PCa in bone and subcutaneous tumors in a preclinical setting. Our data clearly show that cabozantinib treatment kills tumor cells in the bone and in subcutaneous tumors as demonstrated by large necrotic areas in treated tumors, tumor regression, and lower PSA in animals treated with cabozantinib vs. control animals. Interestingly, there are remaining foci of healthy tumor cells adjacent to the necrotic areas, suggesting involvement of angiogenesis and also indicating that the treatment did not constitute a cure, and possibly continuous treatment might be needed to control the disease progression. Notably this was true for androgen-sensitive as well as castration-resistant tumors.

It is important to note that in the clinical situation cabozantinib treatment does not result in consistent decreases of PSA. In many patients PSA levels did not correlate with the reduction in tumor burden that resulted from cabozantinib treatment. Since the observed regression in soft tissue lesions suggests that cabozantinib may have an effect on tumors that is independent of bone environment, this finding is surprising. As such, it is necessary to delineate whether cabozantinib acts on the tumor and/or the microenvironment, and whether this efficacy is a function of androgen sensitivity. Our results agree with the clinical observations. Even though the PSA levels were lower in the treated group vs. the control group, the PSA levels were not lower in the majority of animals bearing intratibial tumors after treatment when compared to pre-treatment levels. Taken together, our data indicate that cabozantinib is efficacious in models of androgen-sensitive PCa as well as in CRPC, and show that cabozantinib efficacy does not solely depend on the bone microenvironment. These data are consistent with cabozantinib’s clinically beneficial observations in patients both with and without bony metastases.

One of our objectives was to investigate the mechanisms of cabozantinib effects on the tumor. Our analysis showed the presence of large necrotic areas in the tumors in bone and significant decreases in the volume of subcutaneous tumors. To demonstrate that the observed inhibitory effects of cabozantinib are due to inhibition of VEGFR2 and MET signaling one would like to demonstrate “on target effects” *in vivo*. However, the levels of VEGFR2 and MET were very low or undetectable in LuCaP 23.1 and C4-2B tumors, and because very little of the tumor tissue was available following cabozantinib treatment, we could not evaluate the on target effects by looking at decreases in phosphorylaiton of these targets. However, this does not indicate that the cabozantinib inhibition of tumor growth does not involve VEGFR2 and MET signaling alterations. It is certainly possible that cabozantinib does have inhibitory effects on the expected kinases even though in these studies, the kinase levels were below the IHC level of detection in the pre-treated xenografts. Even at these low levels, inhibition of these kinases could have a biological effect. As an alternative approach to examine potential mechanisms of cabozantinib effects, we used qPCR. Our results showed decreased levels of survivin and MYC, genes associated with tumor progression, and HIF1A and VEGFR2m, that are associated with tumor progression and angiogenesis in the cabozantinib–treated C4-2B tumors. These qPCR data, the expression of cabozantinib targets in PCa, and cabozantinib’s ability to inhibit multiple kinases, suggest that cabozantinib might affect tumor cells directly as well as indirectly via effects on the tumor microenvironment, and therefore might be effective across a broad spectrum of PCa phenotypes.

The 60 mg/kg dose was well-tolerated in our preclinical models for four weeks of administration when tumors were growing in the bone; but body weight loss was observed with longer treatment. Interestingly the loss of body weight was more pronounced in animals bearing LuCaP 23.1 tumors than in those with C4-2B tumors. A lower dose of cabozantinib (30 mg/kg) was well-tolerated up to six weeks, and the animals lost less weight compared to those that received a higher dose. Therefore, we conclude that cabozantinib treatment is well-tolerated for 4-6 weeks with some negative effects on body weight after prolonged treatment of intra-tibial tumors. 

Our results show that cabozantinib affects not just the tumor, but also the bone response to the tumor. We used two models that cause bone formation when growing in the bone environment: LuCaP 23.1 and C4-2B. Interestingly, we observed opposing effects of cabozantinib on bone volume in these models; large decreases in bone volume in the LuCaP 23.1 tumored tibiae and small increases in bone volume in C4-2B tumored tibiae. We hypothesize that the differences are due to the different magnitude of new bone formation associated with the two different tumors. LuCaP 23.1 is highly osteoblastic, causing 5-fold increases in bone volume, and we hypothesize that the decreases in BV in the LuCaP 23.1 model after cabozantinib treatement are due to cabozantinib’s tumor inhibitory activity (less tumor=less new bone). In comparison to LuCaP 23.1, C4-2B tumors are osteoblastic but cause only ~1.5 fold increase in BV and also induce a significant osteolytic reaction [44]. Therefore, when cabozantinib treatment results in smaller C4-2B tumors, it leads to decreased alteration of bone remodeling caused by tumor (which should result in decreased BV vs untreated tumored tibiae). However, since cabozantinib alters normal bone remodeling and cabozantinib treatment causes increases in BV in normal bone (see below), we hypothesize that the overall increased BV detected is a combination of cabozantinib effects on tumor and normal bone. 

Because VEGFR2 and MET signaling are important in bone biology, it is clear that cabozantinib treatment may not only affect tumored bone, but potentially normal bone as well. Our results show that cabozantinib treatment resulted in increased BV in intact as well as castrated animals. Patients with CRPC are typically on ADT, which causes osteopenia and osteoporosis. Therefore, cabozantinib therapy might provide additional benefits to patients with advanced PCa who are on ADT. The systemic effect of cabozantinib on the skeleton might lead to decreased number of and time to skeletal related events in patients.

In conclusion, we demonstrate that cabozantinib targets are expressed in advanced PCa, indicating that this treatment has the potential for substantial efficacy in the heterogeneous PCa population. Angiogenesis inhibition has not yielded significant advances in the treatment of CRPC to date, and a growing body of evidence suggests that signaling through MET may be a compensatory mechanism by which tumor cells escape anti-angiogenic therapy. Thus, since cabozantinib targets VEGFR2, MET and a number of other RTKs simultaneously, it represents an attractive, new opportunity in anti-angiogenic CRPC treatment. Furthermore, our results show that cabozantinib inhibits tumors in two different PCa: an androgen-dependent osteoblastic model and a castration-resistant mixed osteoblastic/osteolytic model. Furthermore, cabozantinib also affects tumors growing in the bone and subcutaneous tumors, again indicating the potentially high clinical impact of this treatment. 

## Supporting Information

Figure S1 **MET, P-MET, and VEGFR2 immunoreactivity in normal prostate (NP) and primary PCa.** Graphical profiles illustrating distributions of staining intensity calculated as simple averages across all non-missing sections in each staining category. Orange filled circles represent mean staining indices and orange bars indicate 95% CIs. High levels of MET were detected in both NP and PCa; P-MET and VEGFR2 immunoreactivity was minimal. No significant differences were detected in these 3 proteins when comparing NP to PCa.
(TIF)

Figure S2 **Analysis of cabozantinib targets in PCa xenografts.** A. MET, VEGFR2m, AXL, KIT, and RET are expressed at various levels in all 24 xenograft lines tested as determined by qPCR. For this analysis RNA isolated from subcutaneous tumors grown in intact male mice was used. Target expression was normalized to RPL13a. The results were log transformed for statistical analysis. Significance of the differences was evaluated by 2-sided t-test. B. Neuroendocrine LuCaP models (NE, n=4) express higher levels of the cabozantinib targets in comparison to adenocarcinoma models (AD, n=20). C. IHC for MET and P-MET was performed and analyzed. No significant differences were detected in levels of these proteins between LuCaP models with high response to castration (>3 fold survival benefit), low response to castration, or in comparison to neuroendocrine models.
(TIF)

Figure S3 **Q-PCR analyses of C4-2B control and cabozantinib tumors.** RNA was extracted from subcutaneous tumors and qPCR was performed to determine levels of MET, VEGFR2m, ALX and RET. VEGFR2m showed a trend to decrease after cabozantinib treatment, while levels of the other targets were not significantly altered. Our previous qPCR did not detect KIT messages in C4-2B tumors, and therefore we did not include KIT in this analysis. Cabozantinib treatment also resulted in alteration of levels of MYC, endoglin and survivin, indicating effects on tumor. (One should notice that these analyses had only 80% power to detect differences of 60-100% between the groups.).
(TIF)

Table S1 **Sequences of qPCR primers.**
(DOCX)

Methods S1 (DOCX)
