# Correction: Cabozantinib Inhibits Growth of Androgen-Sensitive and Castration-Resistant Prostate Cancer and Affects Bone Remodeling

**DOI:** 10.1371/journal.pone.0106797

**Published:** 2014-08-21

**Authors:** The PLOS ONE Staff

There are some missing funding sources for this article. Please refer to the correct funding statement below:

The studies in this manuscript were funded by Exelixis Inc.; PO1 National Institutes of Health grant (PO1CA085859); Pacific Northwest Prostate Cancer SPORE grant (P50CA097186); Prostate Cancer Foundation; Richard M. Lucas Foundation; and University of Washington Bridge funding. Two of the co-authors (DTA and FS) are employees of Exelixis. They were involved in study design, data analyses, decision to publish and preparation of the manuscript.
