# Supplementary figures and images for: Cabozantinib Inhibits Growth of Androgen-Sensitive and Castration-Resistant Prostate Cancer and Affects Bone Remodeling
Source: PLoS One. 2013 Oct 25;8(10):e78881. doi: 10.1371/journal.pone.0078881 (PMC3808282; doi:10.1371/journal.pone.0078881)

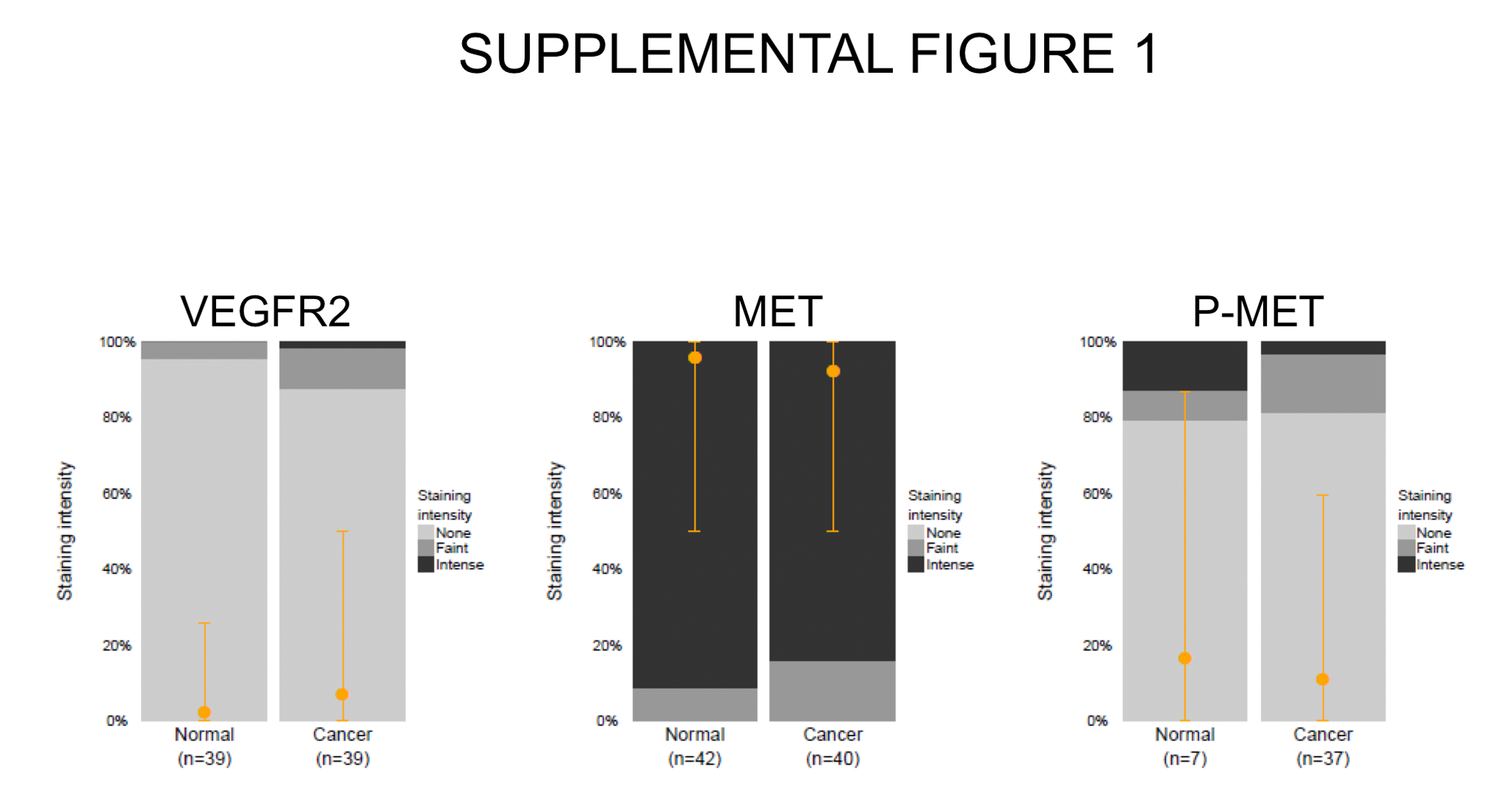

Supplement: Figure S1 — MET, P-MET, and VEGFR2 immunoreactivity in normal prostate (NP) and primary PCa. Graphical profiles illustrating distributions of staining intensity calculated as simple averages across all non-missing sections in each staining category. Orange filled circles represent mean staining indices and orange bars indicate 95% CIs. High levels of MET were detected in both NP and PCa; P-MET and VEGFR2 immunoreactivity was minimal. No significant differences were detected in these 3 proteins when comparing NP to PCa. (TIF) [file pone.0078881.s001.tif]

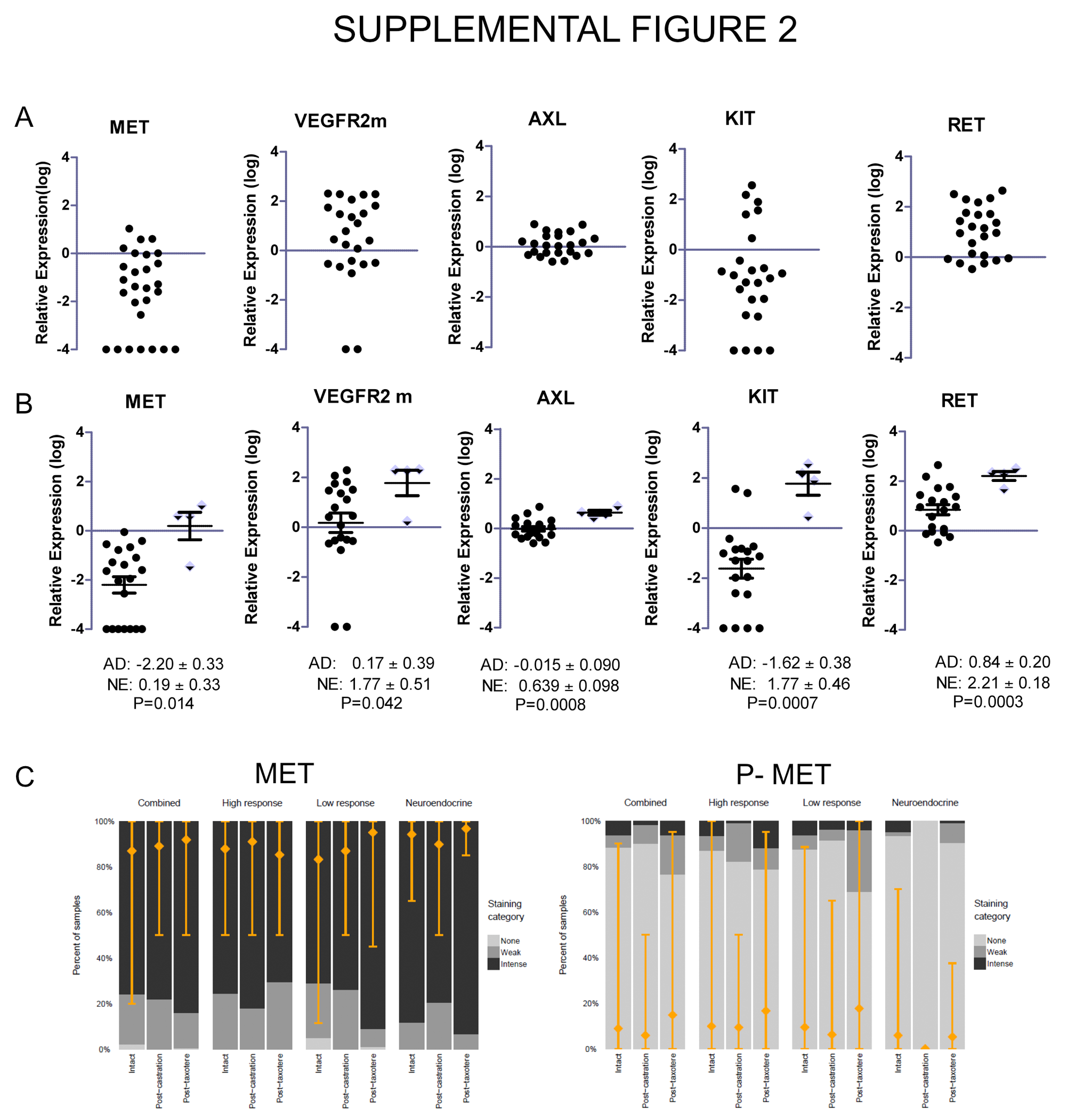

Supplement: Figure S2 — Analysis of cabozantinib targets in PCa xenografts. A. MET, VEGFR2m, AXL, KIT, and RET are expressed at various levels in all 24 xenograft lines tested as determined by qPCR. For this analysis RNA isolated from subcutaneous tumors grown in intact male mice was used. Target expression was normalized to RPL13a. The results were log transformed for statistical analysis. Significance of the differences was evaluated by 2-sided t-test. B. Neuroendocrine LuCaP models (NE, n=4) express higher levels of the cabozantinib targets in comparison to adenocarcinoma models (AD, n=20). C. IHC for MET and P-MET was performed and analyzed. No significant differences were detected in levels of these proteins between LuCaP models with high response to castration (>3 fold survival benefit), low response to castration, or in comparison to neuroendocrine models. (TIF) [file pone.0078881.s002.tif]

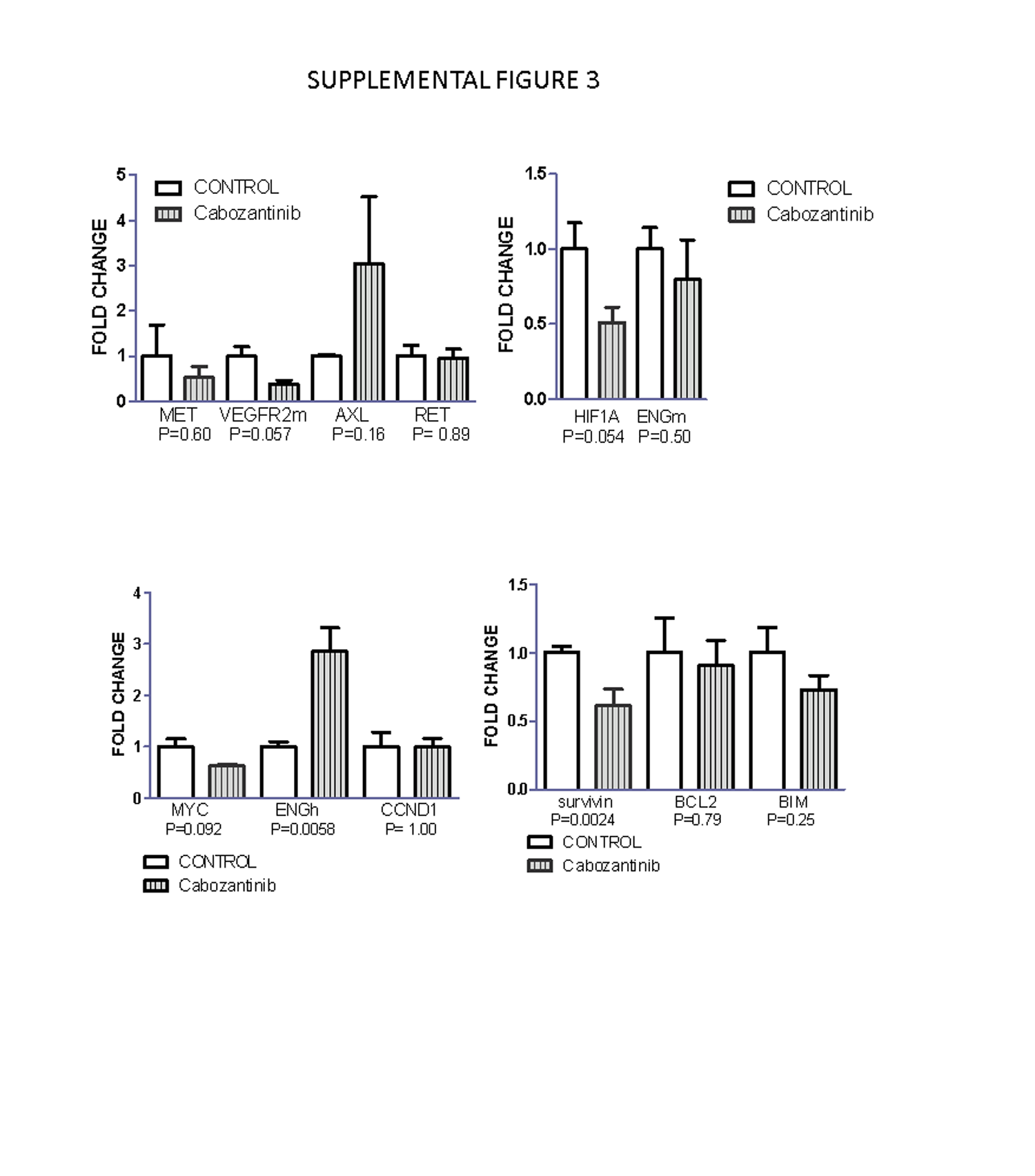

Supplement: Figure S3 — Q-PCR analyses of C4-2B control and cabozantinib tumors. RNA was extracted from subcutaneous tumors and qPCR was performed to determine levels of MET, VEGFR2m, ALX and RET. VEGFR2m showed a trend to decrease after cabozantinib treatment, while levels of the other targets were not significantly altered. Our previous qPCR did not detect KIT messages in C4-2B tumors, and therefore we did not include KIT in this analysis. Cabozantinib treatment also resulted in alteration of levels of MYC, endoglin and survivin, indicating effects on tumor. (One should notice that these analyses had only 80% power to detect differences of 60-100% between the groups.). (TIF) [file pone.0078881.s003.tif]
