# Supplementary material for: Cabozantinib Inhibits Growth of Androgen-Sensitive and Castration-Resistant Prostate Cancer and Affects Bone Remodeling
Source: PLoS One. 2013 Oct 25;8(10):e78881. doi: 10.1371/journal.pone.0078881 (PMC3808282; doi:10.1371/journal.pone.0078881)
Supplement: Table S1 — Sequences of qPCR primers. (DOCX) [file pone.0078881.s004.docx]

Table S1: Sequences of q-PCR primers

| Gene | Forward Primer | Reverse Primer |
| --- | --- | --- |
| MET | CATGCCGACAAGTGCAGTA | TCTTGCCATCATTGTCCAAC |
| AXL | GATGGACAGATCCTGGAGCTCG | GATGCCCATCCCATCGTCTGAC |
| VEGFR2 (m) | SABio Cat #PPM03057A | SABio Cat #PPM03057A |
| VEGFR2 (h) | GTGACCAACATGGAGTCGTG | CCAGAGATTCCATGCCACTT |
| KIT | TGACTTACGACAGGCTCGTG | CCACTGGCAGTACAGAAGCA |
| RET | GTGTGAGTGGAGGCAAGGAG | GTCCTGAGGGCAAATGTTGA |
| Endoglin (h) | AGGCAGAGGACAGGGGTGACAA | GAGGAAGTGTGGGCTGAGGTAGAG |
| Endoglin (m) | GTGTTCCTGGTCCTCGTTTC | GTGGTTGCCATTCAAGTGTG |
| HIF1α (m) | GCACAGAAGCAAAGAACCCATTTTC | GGCAGTGGTAGTGGTGGCATTAG |
| HIF1α (h) | TTACCTTCATCGGAAACTCCAAAGC | ACTGGGACTGTTAGGCTGGGAAAA |
| Survivin | GCTTCATCCACTGCCCCACTGAG | TTTTGTTCTTGGCTCTTTCTCTGTCC |
| cMyc | CACTGGAACTTACAACACCCGAGC | ACCGAGTCGTAGTCGAGGTCATAG |
| RPL13a | CCTGGAGGAGAAGAGGAAAGAG | TTGAGGACCTCTGTGTATTTGTCAA |
