# Supplementary material for: Cabozantinib Inhibits Growth of Androgen-Sensitive and Castration-Resistant Prostate Cancer and Affects Bone Remodeling
Source: PLoS One. 2013 Oct 25;8(10):e78881. doi: 10.1371/journal.pone.0078881 (PMC3808282; doi:10.1371/journal.pone.0078881)
Supplement: Methods S1 — (DOCX) [file pone.0078881.s005.docx]

**Methods S1**

*Real-time PCR*

Real-time PCR was carried out as described previously. Cycling conditions were as follows: 50°C for 2 minutes and 95°C for 2 minutes, followed by 40 cycles at 95°C for 10 seconds and annealing/extension at 60°C for 30 seconds, followed by a final extension at 72°C for 7 minutes.

*Proliferation Assay*

C4-2B and MC3T3 cells (2.4×10^4^ cells/well) were plated in a 6-well plate in MEM + 10% FBS overnight, followed by treatment with 0.01, 0.03, 0.10, 0.30, 1.00, or 3.00 µM cabozantinib for 5 days. Cells were trypsinized and counted with a hemocytometer. Experiments were performed at least three times and carried out in duplicate.

*Mineralization Assay*

MC3T3 cells were seeded in MEM + 10% FBS in six-well plates and allowed to reach confluence, at which point mineralization media was added and cells were treated with 0.01, 0.03, 0.10, 0.30, 1.00, or 3.00 µM cabozantinib. Cells were fixed with 10% formalin at day 11, stained with alizarin red, and absorbance was measured at 450 nm.

*Alkaline Phosphatase Assay (ALP)*

ALP activity was assessed using a colorimetric kit (AbCam, ab83369, Cambridge, MA) according to manufacturer’s directions. MC3T3 cells were seeded (5000 cells/well) in a 24-well plate in MEM + 5% FBS overnight, followed by treatment with 0.01–3.00 µM cabozantinib. ALP activity was assayed at day 2, 4 and 7 and normalized by whole-cell lysate protein concentration.

*ARE reporter Assay*

C4-2B cells were transfected using the Amaxa Nucleofector with solution V and program T-27 according to the manufacturer's recommendations (Amaxa Biosystems, Inc., Gaithersburg, MD) with an ARE reporter and a TK renilla luciferase plasmid and after 48 hrs, a dual-luciferase assay was used as described previously [[1](#_ENREF_1)].

*Statistical Analysis of IHC*

For each core in each TMA, a staining index was constructed as a weighted combination of the 3-point staining intensities, with weights given by the percentage of tissue staining at each intensity. Linear mixed models were fit to the staining index conditional on metastasis location with random effects for each patient or animal. Following evaluation of modeling assumptions and data transformation as necessary, the fitted models were used to quantify differences in immunoreactivity between metastatic locations and to test statistical significance.

The staining index was calculated as follows:

$Index=\frac{0}{2}*None+\frac{1}{2}*Fa\mathrm{int}+\frac{2}{2}*Intense$ (1).

The mean staining indices between tissues types were compared using a linear mixed model:

$\begin{matrix} Index_{ij}=\beta_{0}+\beta_{1}(Tissue)_{ij}+b_{i}+\varepsilon_{ij} \\ \end{matrix}$ (2),

where *i* indexes patients, *j* indexes cores within patients, *Tissue* indicates the metastasis location (0=tissue A and 1=tissue B), *b_i_* is an independently normally distributed patient-specific random effect with mean 0 and variance
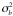
 and *ε_ij_* is an independently normally distributed error term with mean 0 and variance
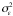
. β*_0_* is the mean staining index in tissue A, and *β_0_+* *β_1_* is the mean staining index of tissue B.

To account for variable lengths of follow-up times and to control for other variables previously shown to be associated with time to recurrence, the following model was additionally fit to staining associations between recurrent and non-recurrent patients:

$\lambda(t)=\lambda_{0}(t)\exp\left\{\theta_{1}Age_{i}+\theta_{2}Gleason_{i}+\theta_{3}Volume_{i}+\theta_{4}Index_{ij}+\tau_{i}\right\}$ (3),

where *λ(t)* is the hazard of recurrence *t* months after radical prostatectomy (RP), *λ_0_(t)* is an unspecified baseline hazard of recurrence, *Age* is patient age at RP, *Gleason* is the Gleason sum at RP, *Volume* is tumor volume at RP, *Index* is the staining index, and *τ_ij_* is an independent normally distributed frailty with mean 0 and variance
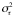
.

*Statistical Analysis of PSA and Tumor Response to Cabozantinib Treatment*

Longitudinal tumor measurements and PSA serum levels were log-transformed and modeled using linear mixed models with random effects for each animal. The models estimated growth rates in cabozantinib or control groups while accounting for variability in the initial measurements across animals. Following standard diagnostic assessment of model fit, we simulated 1000 datasets from each ﬁtted model, calculated empirical mean and 95% confidence limits at each time point, and reﬁt the models to these datasets. Final results represent means and 95% conﬁdence limits of 1000 bootstrap replicates. Specifically, growth trajectories for each variable were predicted by fitting data to the following model:

$\log(Measure_{ij})=\beta_{0}+\beta_{1}Week_{ij}+\beta_{2}Cabozantinib_{ij}+\beta_{3}Week_{ij}*Cabozantinib_{ij}+b_{i}+\varepsilon_{ij}$ (4),

where $\log(Measure_{ij})$ is the log-transformed body weight, PSA, or tumor volume measurement *j* for animal *i*, *Week_ij_* is a week of measurement, *cabozantinib_ij_* indicates treatment group (0=control and 1=cabozantinib), *b_i_* is an animal-specific independent and normally distributed random effect with mean 0 and variance $\sigma_{b}^{2}$, and *ε_ij_* is an independent and normally distributed error term with mean 0 and variance $\sigma_{e}^{2}$.

Supplemental TABLE 1

q-PCR primers

| Gene | Forward Primer | Reverse Primer |
| --- | --- | --- |
| MET | CATGCCGACAAGTGCAGTA | TCTTGCCATCATTGTCCAAC |
| AXL | GATGGACAGATCCTGGAGCTCG | GATGCCCATCCCATCGTCTGAC |
| VEGFR2 (m) | SABio Cat #PPM03057A | SABio Cat #PPM03057A |
| VEGFR2 (h) | GTGACCAACATGGAGTCGTG | CCAGAGATTCCATGCCACTT |
| KIT | TGACTTACGACAGGCTCGTG | CCACTGGCAGTACAGAAGCA |
| RET | GTGTGAGTGGAGGCAAGGAG | GTCCTGAGGGCAAATGTTGA |
| Endoglin (h) | AGGCAGAGGACAGGGGTGACAA | GAGGAAGTGTGGGCTGAGGTAGAG |
| Endoglin (m) | GTGTTCCTGGTCCTCGTTTC | GTGGTTGCCATTCAAGTGTG |
| HIF1α (m) | GCACAGAAGCAAAGAACCCATTTTC | GGCAGTGGTAGTGGTGGCATTAG |
| HIF1α (h) | TTACCTTCATCGGAAACTCCAAAGC | ACTGGGACTGTTAGGCTGGGAAAA |
| Survivin | GCTTCATCCACTGCCCCACTGAG | TTTTGTTCTTGGCTCTTTCTCTGTCC |
| cMyc | CACTGGAACTTACAACACCCGAGC | ACCGAGTCGTAGTCGAGGTCATAG |
| RPL13a | CCTGGAGGAGAAGAGGAAAGAG | TTGAGGACCTCTGTGTATTTGTCAA |
